# Adeno-associated virus (AAV)-mediated suppression of Ca2+/calmodulin kinase IV activity in the nucleus accumbens modulates emotional behaviour in mice

**DOI:** 10.1186/1471-2202-8-105

**Published:** 2007-12-03

**Authors:** Miriam Schneider, Rainer Spanagel, Sheng-Jia Zhang, Hilmar Bading, Matthias Klugmann

**Affiliations:** 1 Central Institute of Mental Health (ZI), Department of Psychopharmacology, J5, 68159 Mannheim, Germany; 2 Department of Neurobiology, Interdisciplinary Center for Neurosciences (IZN), University of Heidelberg, Im Neuenheimer Feld 364, 69120 Heidelberg, Germany; 3 Department of Physiological Chemistry, University of Mainz, Duesbergweg 6, 55099 Mainz, Germany

## Abstract

**Background:**

Calcium/calmodulin-dependent protein kinase IV (CaMKIV) controls activity-dependent gene transcription by regulating the activity of the cyclic AMP response element binding protein (CREB). This signaling pathway is involved in gating emotional responses in the CNS but previous studies did not address the potential roles of CaMKIV in discrete brain regions. In the present study, we aimed at specifically dissecting the role of CaMKIV in the nucleus accumbens of adult mice.

**Results:**

We used recombinant adeno-associated virus (rAAV)-mediated gene transfer of a dominant-negative CaMKIV variant (rAAV-dnCaMKIV) to inhibit endogenous CaMKIV in the nucleus accumbens. rAAV-dnCaMKIV treated animals were subjected to a battery of tests including, prepulse inhibition of the acoustic startle response, open field, social interaction and anxiety-related behaviour. We found that basal locomotor activity in the open field, and prepulse inhibition or startle performance were unaltered in mice infected with rAAV-dnCaMKIV in the nucleus accumbens. However, anxiogenic effects were revealed in social interaction testing and the light/dark emergence test.

**Conclusion:**

Our findings suggest a modulatory role of CaMKIV in the nucleus accumbens in anxiety-like behaviour but not sensorimotor gating.

## Background

Long-lasting neuroadaptations in mesolimbic structures, particularly in the nucleus accumbens (NAc), influence behavioural responses to emotional stimuli. This experience-based behaviour occurs as a result of activity-dependent synaptic plasticity. At the molecular level, electrical activation of neurons leads to opening of ligand and/or voltage-gated calcium channels and generates intracellular calcium transients [1]. Calcium signals can propagate to the cell soma, invade the cell nucleus, and lead to the activation of the nuclear calcium/calmodulin-dependent kinase IV (CaMKIV) [2,3]. CaMKIV activates several transcription factors such as ATF-1, MEF2D and NF-kappaB [4-6], and is a key regulator of neuronal gene expression that stimulates transcription through the phosphorylation of the cAMP response element binding protein (CREB) and activation of the CREB co-activator, CREB binding protein (CBP) [2,3,7].

The activation of CREB after exposure to emotional stimuli has been shown to alter gating between these environmental stimuli and their behavioural responses [8]. Disruption of CREB function within the NAc increases anxiety-related behaviour while CREB overexpression reduces anxiogenic responses under certain conditions [9,10]. In line with these findings, NAc-specific expression of the inducible cAMP early repressor (ICER), a natural inhibitor of CREB-mediated transcription, has been shown to increase measures of anxiety in the elevated plus maze and neophobia to novel tastes [11]. Two independent CaMKIV-deficient mouse lines were generated that showed overlapping phenotypes. Means and coworkers demonstrated that the targeted disruption of the CaMKIV gene results in impaired cerebellar LTD and motor control [12] while the other knockout strain [13] had mild cerebellar abnormalities and deficits in long-term potentiation (LTP). The latter mutants also exhibited decreased fear memory [14] and reduced anxiety-like behaviour in the elevated plus maze and dark-light emergence test [15]. Functional redundancy and/or effects during development are inherent problems that can complicate the interpretation of results obtained in knockout mice and may mask the roles of CaMKIV in defined brain regions. To study the role of CaMKIV in cognitive processes, Kang and colleagues generated transgenic mice expressing a dominant-negative (dn) form of CaMKIV in the postnatal forebrain [16]. These mice showed normal locomotor and emotional behaviour. Although this study revealed insight into the role of CaMKIV in the context of complex behaviour, it did not allow the characterization of CaMKIV function specifically in the NAc of adult animals. To investigate this, we used a recombinant adeno-associated virus (rAAV) gene transfer system to interfere with CaMKIV function specifically in the NAc. CaMKIV activity was suppressed by rAAV-mediated expression of a kinase-dead mutant of CaMKIV [17]. The results obtained indicate that NAc-specific suppression of CaMKIV activity increased anxiety-related behaviour whereas sensorimotor gating was unaffected.

## Results

### rAAV-mediated gene transfer

**Figure 1:**
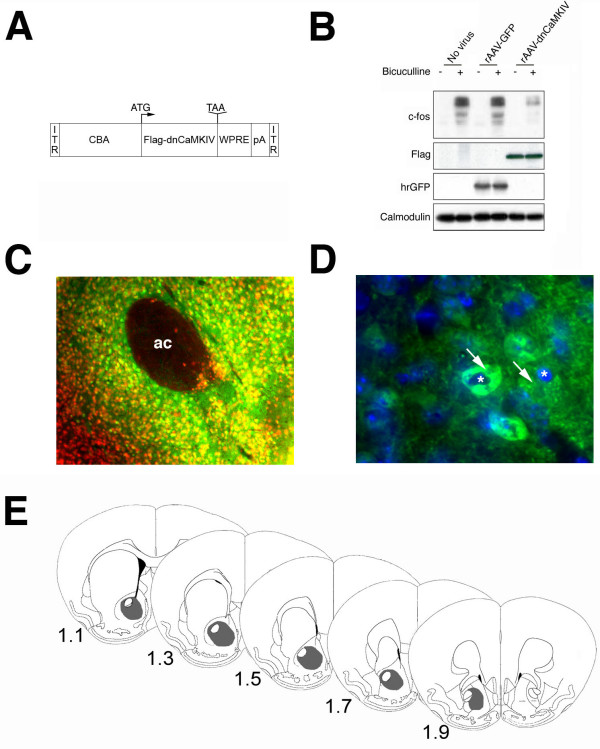
**Characterization of rAAV-mediated transgene expression**. A) Schematic illustration of the rAAV-dnCaMKIV expression cassette, for nomenclature see Methods section. B) Immunoblot analysis of neuronal activity-dependent induction of c-Fos expression in uninfected cultured mouse hippocampal neurons or in cultured mouse hippocampal neurons infected with rAAV-dnCaMKIV or rAAV-hrGFP. The neurons were treated for 4 hr with the GABA_A _receptor bicuculline (50 μM) to induce action potential bursting [2, 19], or were left untreated. Expression of c-Fos, hrGFP, Flag-tagged dnCaMKIV, and calmodulin (loading control) was analyzed. C) Immunohistochemical analysis of dnCaMKIV expression (using antibodies to the Flag-tag) and expression of the neuronal marker NeuN in the NAc of animals infected in the NAc with rAAV-dnCaMKIV. The overlay of representative photomicrographs (10× objective) of Flag-immunohistochemistry (green) and NeuN (red) is shown. ac, anterior commissure. D) Photomicrograph (100× objective) showing overlay of Flag-immunoreactivity (green) and Hoechst stain (blue). Arrows indicate the cytosolic localization of Flag-tagged dnCaMKIV in two representative neurons; the nuclei in those neurons are indicated with asterisks. E) Schematic diagrams showing the approximate extension of transgene expression (gray shading) in rAAV-dnCaMKIV injected mice. Numbers, distance from bregma [42].

We generated a rAAV vector expressing Flag-tagged dominant-negative CaMKIV under the control of the cytomegalovirus enhancer/chicken beta actin (CBA) promoter (Fig. 1A). As control vectors, we packaged the rAAV-plasmid without any coding sequence (rAAV-empty), or with the humanized renilla green fluorescent protein open reading frame (rAAV-hrGFP). We have previously shown that the dnCaMKIV mutant inhibts CRE-mediated transcription in AtT20 cells [18]. In the present study, we first assessed the efficacy of gene transfer and expression of rAAV-dnCaMKIV in primary hippocampal neurons by immunoblot analysis and confirmed the functionality of the dnCaMKIV vector. Figure 1B illustrates that rAAV-dnCaMKIV but not rAAV-hrGFP dramatically reduced expression of the CREB-target gene c-fos after induction of action potential (AP) bursting. The transgenes were readily detected using specific antibodies indicating a high transduction efficiency as described previously [19].

Next, the vectors were injected bilaterally into the NAc of adult mice. rAAV-mediated gene expression requires at least three weeks to peak in the rodent brain and then persists at stable levels without overt inflammation or immunogenicity [20]. Three weeks after surgery, the transduction efficiency was assessed by Flag-immunohistochemistry. In line with the known neurotropism of chimeric rAAV1/2 vectors [21], robust transgene expression was found in NAc neurons in all rAAV-dnCaMKIV-treated animals (Fig. 1C). As expected [22], Flag immunoreactivity localized to the cytosol of transduced cells (Fig. 1D). Similar to our previous studies using rAAV1/2 as gene delivery system in the NAc [23-25], the transduction covered most of the NAc core and shell subregions and was restricted approximately 1 mm around the injection site in the NAc (Fig. 1E). Next, the response of dnCaMKIV expressing animals on emotional stimuli was investigated in a battery of behavioural tests.

### Locomotor activity

The locomotor activity of all animals was assessed in an open field. Suppression of CaMKIV activity did not affect basal locomotor activity. Animals expressing dnCaMKIV did not differ significantly from control-infused rAAV-empty mice (emtpy) in activity time [s] (Values ± S.E.M.: dnCaMKIV: 933.9 ± 18.1; EMPTY: 973.2 ± 20.8; Student's t-test, p > 0.05), distance travelled [cm] (Values ± S.E.M.: dnCaMKIV: 6018.1 ± 259.6; EMPTY: 6654.5 ± 275.4; Student's t-test, p > 0.05) and rearing (Values ± S.E.M.: dnCaMKIV: 237.9 ± 21.7; EMPTY: 262.1 ± 26.4; Student's t-test, p > 0.05).

### Light/dark emergence test

**Figure 2:**
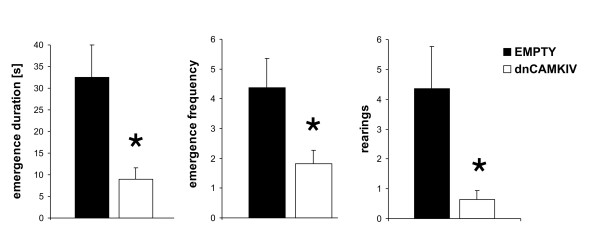
**Anxiety-related behaviours observed in the dark/light box**. Expression of dnCaMKIV in the NAc increased anxiogenic behaviour during dark/light emergence test performance. Time spent in the lit compartment, emergence frequency and rearing were significantly reduced in dnCaMKIV animals compared to rAAV-empty infused controls (EMPTY) (dnCaMKIV: n = 11, EMPTY: n = 11; p < 0.05 is indicated by asterisks). Means ± S.E.M. are shown.

To investigate anxiety-related behaviour, a classical anxiety paradigm, the light/dark emergence test, was chosen. Expression of dnCaMKIV in the NAc significantly increased anxiety-related behaviours in the light/dark emergence test. Frequency of emergence into the lit compartment, time spent there (duration) [s] and rearing were decreased compared to controls (Fig. 2) (Student's t-test, p < 0.01; p = 0.03; p = 0.02 respectively). No effects were seen on emergence latency and risk assessment (data not shown, Student's t-test, p > 0.05).

### Social interaction test

**Figure 3:**
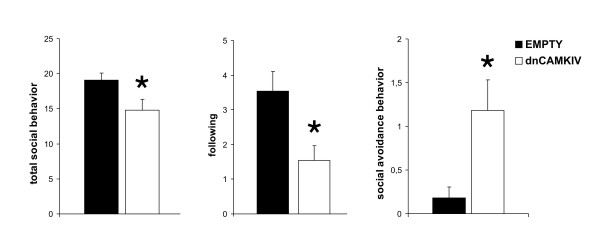
**Social interaction testing**. Interaction with an unknown social partner revealed an increase in anxiety-related behaviour in rAAV-dnCAMKIV treated animals. The total amount of social behaviours was decreased in those mice, mainly due to a significant decrease in approaching and following the social partner. Social avoidance/anxiety-related behaviour (stretched-attend posture and evade upon contact) was increased in animals expressing dnCaMKIV compared to EMPTY (dnCaMKIV: n = 11, EMPTY: n = 11; p < 0.05 is indicated by asterisks). Means ± S.E.M. are shown.

To assess the effects of dnCaMKIV expression on different aspects of anxiety-related behaviour and social behaviour animals were tested in a social interaction paradigm. Compared to controls, rAAV mediated dnCaMKIV expression in the Nac significantly reduced the total amount of social behaviour in the social interaction test (Student's t-test, p = 0.03). This decrease in social behaviour was mainly related to a reduction in following behaviour observed in these animals. Furthermore, expression of dnCaMKIV in the NAc significantly increased anxiety-like behaviour in the social interaction test (Fig. 3) (Student's t-test, p = 0.01). No significant effects were found for contact behaviour, social exploration or self grooming (data not shown, Student's t-test, p > 0.05).

### Prepulse inhibition (PPI) of the acoustic startle response (ASR)

To further investigate potentially increased anxiety responses and difficulties in attentional processing or sensorimotor gating, animals were tested for their PPI of the ASR. Animals expressing dnCaMKIV in the NAc did not differ significantly from controls in their ASR magnitudes (Student's t-test, p > 0.05), nor in their PPI performance (ANOVA: F_1,40 _= 0.7, p > 0.05) (Table 1).

**Table 1:** PPI of the ASR.

**Treatment**	**dnCAMKIV**	**EMPTY**
**ASR **± S.E.M.	121.0 ± 18.2	143.4 ± 17.7
**PPI [%] **± S.E.M.		
**Prepulse 72 dB**	26.3 ± 5.4	24.6 ± 9.1
**Prepulse 76 dB**	54.1 ± 3.8	62.9 ± 4.2
**Prepulse 80 dB**	66.3 ± 3.2	74.3 ± 2.5

Suppression of CaMKIV activity in the NAc did not significantly affect PPI or startle magnitude (dnCaMKIV: n = 11, EMPTY: n = 11).

## Discussion

In the present study, we show that expression of a negative interfering mutant of CaMKIV in the NAc of adult mice results in increased anxiety-like behaviour. The behavioural abnormalities in rAAV-dnCaMKIV treated animals appear to be very selective since locomotor activity in the open field, and functional sensorimotor gating assessed by normal PPI performance of the acoustic startle reflex were found unchanged. The social interaction test revealed an increased anxiogenic response towards an unknown social partner in animals expressing rAAV-dnCaMKIV, while other social behavioural elements such as self-grooming, contact behaviourand social exploration did not differ from controls.

Two different approaches were used previously to study the loss of function phenotype of CaMKIV in the brain. Kang and colleagues generated transgenic mice expressing a negative interfering mutant of CaMKIV in the postnatal forebrain [16]. These mice had deficits in long-term memory but showed normal behaviour in the open field or elevated-plus maze. Results obtained by the latter test suggested normal fear-related behaviour in these mutants. In contrast, in mice with a targeted null mutation of the endogenous CaMKIV gene anxiolytic effects were found in the elevated-plus maze and the dark/light emergence test [15]. These authors also reported unchanged open field performance but a reduction of anxiety-like behaviour in the dark-light emergence test. However, in the current study, we observed an increase using the same test. It might not appear surprising that the different approaches to genetically altering CaMKIV function yield non-overlapping or even partially opposing phenotypes. CaMKIV is normally expressed during development and in the adult in many regions of the central nervous system and in the periphery [26,27]. Expression domains in the brain include neocortex, hippocampus, striatum and amygdala, the latter region has been implicated in fear memory. Hence, anxiety-related behavioural responses have been correlated with the role of CaMKIV in the amygdala [14]. In the present study we detected an anxiogenic phenotype after NAc-specific suppression of CaMKIV function. Although the NAc is not considered a key structure for anxiety-related responses, there is increasing evidence for an involvement of this brain region in stress, anxiety and emotional behaviour [9,10]. Furthermore, the ventral striatum (NAc and olfactory tubercle) and the striatum have also been implicated in anxiety-like behaviour [28,29]. The NAc has been characterized as a limbic-motor interface, which refers to the importance of this structure in the integration of different brain circuits, mediating the transfer from motivation/emotion into action [30,31]. These integrating characteristics of the NAc for different brain structures, such as the amygdala, prefrontal cortex, hippocampus and hypothalamus, may underlie its involvement in anxiety-related processes.

The studies addressing anxiety-related behaviour in relation to activity-dependent gene expression in the NAc focused on specific alterations mediated by manipulations of the NAc shell [9-11], while in the present study we targeted CaMKIV activity in both NAc core and shell. It is has been shown that the NAc core is critical for mediating the ability of environmental cues with learned relevance to stimulate and guide behaviour, whereas the NAc shell is more involved in modulating unconditioned behaviours, such as feeding and drug reward. Albeit their different functional roles, it is important to note that the shell and core regions of the NAc are thought to be part of two closely direct interacting networks [32]. It has been suggested that for example cues associated with drug reward through the shell may affect instrumental performance by output of the core [33]. Therefore, these two subregions and their associated circuitry are thought to have a strong influence on each other by specific, possibly GABA-mediated shell-to-core and reciprocal projections [32].

We addressed the consequences of suppression of CaMKIV function exclusively in the NAc of adult mice. Our results differed from those obtained with CaMKIV mouse mutants generated using conventional transgenic technologies. The interpretation of results observed in complete null mutants is inherently complicated due to potential compensatory effects. In addition determining the function of genes in discrete brain regions is difficult because more than one neural circuit is likely to be affected by a genetic deletion in the germ line. The transgenic mouse line overexpressing a negative interfering mutant of CaMKIV in the cortex, hippocampus and striatum after birth was informative but did not allow dissection of CaMKIV function in specific brain areas after completion of neuronal maturation.

We have shown that inhibition of endogenous CaMKIV activity attenuated Ca^2+^-induced expression of c-fos (Fig. 1B) and increases anxiety-like behaviour after viral transfer to the NAc. Likewise, the inhibition or deletion of CaMKIV causes a lack of activity-dependent phosphorylation of CREB at serine 133 and c-fos expression *in vitro *and *in vivo *and behavioural abnormalities [12,16,34]. These data suggest that CaMKIV-dependent activation of CREB/CRE signaling pathways is involved in complex behaviour. Support for the hypothesis that increased anxiety-like behaviour found in the rAAV-dnCaMKIV treated animals might be linked to acute reduction of CREB/CRE-dependent signaling in the NAc comes from a study in which herpes simplex virus-mediated expression of dominant-negative mutant of CREB in the NAc of adult mice and rats enhanced anxiogenic and aversive behavioural responses to emotional stimuli while overexpression of CREB had the opposite effect [9,10]. Furthermore, induction of the endogenous CREB antagonist, ICER, in the NAc has been shown to increase measures of anxiety in the elevated plus maze and neophobia to novel tastes [11]. Barrot et al. (2005) showed that inhibition of CREB in the NAc affected the initiation of sexual behaviour which was associated with an anxiety-like phenotype. In these animals the latency for the first mount as well as for intromission was largely increased without affecting later copulatory parameters (such as ejaculation and the number of mounts to reach ejaculation) and could be restored by treatment with the anxiolytic drug diazepam, suggesting an anxiogenic response upon the initial contact with the female [10]. Similarly, the deficits in the social behaviour of rAAV-dnCaMKIV mice analyzed in this study may mainly derive from an increase in anxiety-related responses (increased stretched-attend posture, evade upon contact and a decrease in following) towards the unknown social partner that leaves unaffected other social behaviours such as exploration or contact behaviour.

## Conclusion

Our data show that the expression of a dnCaMKIV in the NAc induces an increase in anxiety-related behaviour similar to that observed after overexpression in the NAc of either ICER or a dominant-negative version of CREB (mCREB) [9-11]. In addition to anxiety, CREB dysregulation in the NAc has been implicated in depression and depressive symptoms [35,36], indicating that understanding the role of CaMKIV in this brain structure is of clinical importance. CREB/CBP has been suggested to be a key regulator of the reactivity of brain "reward" circuits which regulates individual sensitivity to emotional stimuli in general [8]. CaMKIV is an important upstream activator of CREB/CBP-dependent gene transcription, and intracellular signaling through the nuclear calcium-CaMKIV-CREB/CBP pathway might exert a similar or even a subsidiary modulatory influence on emotional gating processes in the NAc. Future virus-based studies will shed light on the role of CaMKIV for the behavioural responses to emotional stimuli and might offer new insights in the pathophysiology of mood-related disorders.

## Methods

### Subjects

Twenty-two naive adult male C57Bl/6NCrl mice (Charles River, Sulzfeld, Germany) weighing 25–30 g were used for this study. Initially all animals were group housed in groups of four under standard conditions in Macrolon cages (Typ II) on a 12 h light-dark schedule (lights on 7:00–19:00). One week before behavioural testing was started, mice were single housed to avoid whisker barbering [37,38]. They had free access to tap water and were fed *ad libitum*. The experiments were done in accordance with the ethical guidelines for the care and use of laboratory animals for experiments, and were approved by the local animal care committee (Karlsruhe, Germany).

### Generation of recombinant adeno-associated virus (rAAV)

A cDNA encoding the dominant-negative mutant of human CaMKIVK75E [17] fused to the coding region of the Flag-tag (generous gift of Anthony R. Means), was subcloned in an AAV plasmid backbone containing the 1.1 kb CMV enhancer/chicken β-actin (CBA) promoter, the woodchuck post-transcriptional regulatory element (WPRE) and the bovine growth hormone polyA (bGH) to yield the construct pAAV-dnCaMKIV. The same pAAV-CBA-WPRE-bGH backbone carrying no cDNA (pAAV-empty) or hrGFP (pAAV-hrGFP) were used as controls [21,39]. rAAV mosaic vectors containing a 1:1 ratio of AAV1 and AAV2 capsid proteins with AAV2 inverted terminal repeats (ITRs) were generated by crosspackaging as described [40]. Briefly, HEK293 cells were transfected with the AAV cis plasmid, the AAV1 and AAV2 helper plasmids and the adenovirus helper plasmid by standard calcium phosphate transfection methods. 48 h after transfection, cells were harvested and the vector purified using heparin affinity columns (Sigma, St. Louise, MO). Genomic titers were determined using the ABI 7300 real time PCR cycler (Applied Biosystems) with primers designed to bGH.

### rAAV vector administration

Briefly, mice were anaesthetised (Fentanyl [0.005 mg/kg]/Domitor [0.15 mg/kg]/Dormicum [2.0 mg/kg] i. p.) and 1 μl of either rAAV-empty or rAAV-dnCaMKIV (3 × 10^11 ^viral genomes/ml) was injected bilaterally into the NAc (+1.4 mm AP, ± 0.9 mm ML, -4.5 mm DV from bregma) using a stereotaxic frame (Kopf Instruments, Tujunga, CA). Vectors were infused at a rate of 200 nl/min using a microprocessor controlled mini-pump (World Precision Instruments, Sarasota, FA). Anesthesia was antagonized using Narcanti (0.12 mg/kg)/Antisedan (0.75 mg/kg)/Anexate (0.2 mg/kg) i. p. Behavioural training began three weeks after vector infusion when transgene protein expression has peaked to remain at stable levels [41].

### Immunohistochemistry and verification of transduction

The brains of all animals were assessed for transgene expression at the end of behavioural testing. Immunostaining of brain sections was done as described [39]. Briefly, mice were killed by transcardiac perfusion under deep anesthesia (pentobarbital). After perfusion with 0.9% NaCl, brains were fixed *in situ *with 10% buffered neutral formalin, pH 7.4 (Sigma-Aldrich, Taufkirchen, Germany). Brains were removed and post-fixed overnight in the same fixative before cryoprotection in 30% sucrose/PBS. Coronal sections (40 μm) were cut using a cryostat. Free-floating sections were rinsed with PBS containing 0.2% Triton-X100 (PBS-Triton), blocked in immunobuffer (4% horse serum in PBS, pH 7.4, with 0.4% Triton X-100) for 30 min, followed by overnight incubation with rabbit anti-Flag (1:1000; Sigma) and mouse anti-NeuN (1:1000; Sigma). Following three washes, sections were incubated with cy3-labeled goat anti-mouse antibodies (Jackson Immunochemical Laboratories, Bar. Harbor, ME) or Alexa488-labeled donkey anti-rabbit Alexa488 antibodies (1:1000; Invitrogen, Karlsruhe, Germany). Before the third wash, the nuclear dye Hoechst 33258 (Invitrogen) was administered for 5 min and fluorescence was visualized using a Zeiss Axiophot microscope. Flag-immunostaining was restricted to the NAc [42] in all rAAV-dnCaMKIV injected animals. There is no protein expressed from the empty vector cassette but spread of rAAV1/2 vectors assessed by in situ mRNA detection is comparable for titer-matched preparations regardless of the transgene [43]. We could detect the needle track in the NAc of all rAAV-empty treated animals in the NAc. All rAAV-injected animals were included in the analyses.

### Cell culture and Immunoblot analysis

Primary hippocampal neurons from new-born C57Black mice were cultured in Neurobasal media (Invitrogen, Gaithersburg, MD, USA) containing 1% rat serum, B27 (Invitrogen, Gaithersburg, MD, USA), and penicillin and streptomycin (Sigma). The procedure used to isolate and culture hippocampal neurons has been described [44,45].

Neurons were infected with rAAVs after 4 days *in vitro *(DIV) and stimulations were done at 10–12 DIV as described [19]. Immunoblotting was done using standard procedures. Protein samples prepared from uninfected and rAAV-infected hippocampal neurons were separated by SDS-PAGE, transferred onto nitrocellulose, and probed with antibodies to the Flag-tag (Sigma), c-Fos (Sigma), calmodulin (Upstate, Charlottesville, VA), and hrGFP (Stratagene). HRP-labeled secondary antibodies were detected using chemiluminescence

### Behavioural testing

Behavioural testing was conducted in all animals (n = 11, for both treatment groups) in the order listed below. Animals were left undisturbed for at least 5 days between the different test sessions. The experimenter was blind to the treatment of the animals.

### Locomotor activity

Locomotor activity was measured in an infrared-beam operated open field (TruScan, Coulbourne Instruments, USA) for 30 min. At the beginning of the test session, each mouse was placed in the middle of the open field. The number of rearings, activity time [s] and distance travelled [cm] were recorded.

### Light/dark emergence test

The emergence test took place in a plastic box (45 × 20 × 25 cm) which consisted of two different compartments separated by a dividing wall with a hole in the centre that allows the animals free access to both sides. The first compartment, with black walls could be closed by a lid and was used as start box. The second compartment had white walls and was bright illuminated (300 lux). Mice were initially placed in the dark, closed compartment and their behaviour was recorded for 5 min. Subsequent video analysis scored the latency of mice to emerge from the dark compartment into the light compartment, the emergence frequency, the duration of time spent in the light compartment, the amount of rearings, and risk assessment behaviour (only head or forepaws are placed in the lit compartment without concomitant movement of the hindlimbs, even if the mouse subsequently entered the area). The apparatus was thoroughly cleaned with 70% ethanol between the sessions.

### Social interaction test

Social interaction was assessed in an open field. The animals were allowed to explore the test arena freely for 2 min before the social partner (male juvenile mouse, 6 weeks of age) was presented for 5 min. The following behavioural elements were quantified. (A) Social behaviour: contact behaviour (grooming, crawling over), social exploration (anogenital and non-anogenital investigation) and approach/following were scored as social behaviours; (B) social avoidance/anxiety-related behaviour: Evade upon social contact and occurrence of stretched-attend posture were scored as anxiety-like responses; (C) self grooming behaviour. The apparatus was thoroughly cleaned with 70% ethanol between the sessions.

### Prepulse inhibition of the acoustic startle reflex

Startle testing occurred in a startle chamber (SR-LAB; San Diego Instruments, San Diego, USA). A loudspeaker inside the box produced a continuous background noise of 68 dB sound pressure level (SPL) as well as the acoustic startle pulses. A white noise pulse was used as the startle stimulus, with an intensity of 120 dB SPL and duration of 40 ms; three different white noise intensities (72, 76 and 80 dB SPL, duration 20 ms) were used as prepulses. An acclimatisation time of 5 min, during which the mice received no stimulus except the background noise, was followed by the presentation of 5 initial startle stimuli. After this habituation program the test program was started with seven different trial types presented in a pseudorandom order: 1.trial: pulse alone, 2.trial: control (no stimulus), 3.trial: pulse with preceding prepulse (prepulse 72 dB SPL 100 ms before pulse), 4.trial: pulse with preceding prepulse (prepulse 76 dB SPL 100 ms before pulse), 5.trial: pulse with preceding prepulse (prepulse 80 dB SPL 100 ms before pulse), 6.trial: prepulse alone (80 dB). A total of 10 presentations of each trial type was given with an interstimulus interval randomized between 10 and 20 s.

### Statistical analysis

PPI was calculated as the per cent decrease of the ASR magnitude in trials when the startle stimulus was preceded by a prepulse [100 × (mean ASR amplitude on pulse alone trials – mean ASR amplitude on prepulse-pulse trials)/mean ASR amplitude on pulse alone trials].

Differences between the treatment groups for all three prepulse intensities were evaluated using a two-way repeated measure ANOVA, followed by post-hoc Tukey *t*-tests for pairwise comparison. The effects of dnCaMKIV on mean locomotor activity, ASR, social interaction and emergence test behaviour were evaluated by Student's *t*-tests. Means + S.E.M are given. p < 0.05 was considered significant.

## Authors' contributions

MK designed and carried out the AAV work, guided and performed stereotaxic surgery, coordinated the study and drafted the manuscript. MS designed and carried out the behavioural experiments, performed stereotaxic surgery, and drafted the manuscript. RS participated in the coordination of the study and contributed to the preparation of the manuscript. SJZ carried out AAV work and the immunoblot. HB initiated the study on nuclear calcium/CaMKIV signalling in NAc functions and contributed to the preparation of the manuscript. All authors read and approved the final manuscript.
